# Imbalances in serum angiopoietin concentrations are early predictors of septic shock development in patients with post chemotherapy febrile neutropenia

**DOI:** 10.1186/1471-2334-10-143

**Published:** 2010-05-28

**Authors:** Brunna E Alves, Silmara AL Montalvao, Franciso JP Aranha, Tania FG Siegl, Carmino A Souza, Irene Lorand-Metze, Joyce M Annichino-Bizzacchi, Erich V De Paula

**Affiliations:** 1 Hematology and Hemotherapy Center, University of Campinas, Campinas, SP, Brazil; 2 Faculty of Medical Sciences, University of Campinas, Campinas, SP, Brazil

## Abstract

**Background:**

Febrile neutropenia carries a high risk of sepsis complications, and the identification of biomarkers capable to identify high risk patients is a great challenge. Angiopoietins (Ang -) are cytokines involved in the control microvascular permeability. It is accepted that Ang-1 expression maintains endothelial barrier integrity, and that Ang-2 acts as an antagonizing cytokine with barrier-disrupting functions in inflammatory situations. Ang-2 levels have been recently correlated with sepsis mortality in intensive care units.

**Methods:**

We prospectively evaluated concentrations of Ang-1 and Ang-2 at different time-points during febrile neutropenia, and explored the diagnostic accuracy of these mediators as potential predictors of poor outcome in this clinical setting before the development of sepsis complications.

**Results:**

Patients that evolved with septic shock (n = 10) presented higher levels of Ang-2 measured 48 hours after fever onset, and of the Ang-2/Ang-1 ratio at the time of fever onset compared to patients with non-complicated sepsis (n = 31). These levels correlated with sepsis severity scores.

**Conclusions:**

Our data suggest that imbalances in the concentrations of Ang-1 and Ang-2 are independent and early markers of the risk of developing septic shock and of sepsis mortality in febrile neutropenia, and larger studies are warranted to validate their clinical usefulness. Therapeutic strategies that manipulate this Ang-2/Ang-1 imbalance can potentially offer new and promising treatments for sepsis in febrile neutropenia.

## Background

Despite improvements in supportive care, sepsis remains the most common cause of death in intensive care units, with mortality rates of 30% to 50 [1,2]. Recently, therapies targeting elements from the inflammatory and coagulation cascades yielded disappointing results in clinical trials [3-5], possibly due to redundancy within the inflammatory pathways activated during sepsis. Endothelial barrier disruption plays a key role in the pathogenesis of sepsis and septic shock, making it an attractive target for studies aimed to identify new therapeutic targets for sepsis [6]. Recently, the participation of VEGF, a vascular growth factor with potent microvascular permeability functions, in the pathogenesis of septic shock was demonstrated [7]. Another important regulator of endothelial barrier function are the angiopoietins (Ang) -1 and -2, and the tyrosine kinase receptor Tie2 expressed in endothelial cells. Binding of Ang-1 to Tie2 maintains the quiescent resting state of the endothelium and reduces vascular permeability in response to inflammatory stimuli. In contrast, Ang-2 inhibits biding of Ang-1 to Tie2, resulting in vessel destabilization [8-10]. Circulating levels of Ang-1 and Ang-2 have been recently evaluated in patients with sepsis, and levels of Ang-2 have been correlated with sepsis severity in children [11] and adults [12-15], when evaluated in patients admitted to intensive care units with established signs and symptoms of sepsis.

Febrile neutropenia (FN) in patients with hematologic malignancies is characterized by increased susceptibility to sepsis complications, and a higher risk of septic shock, with mortality ranging from 2-21% [16]. Most patients with FN are well at the time of fever onset. However, ways to predict the development of fulminant sepsis and septic shock is a great challenge in the care of these patients [17,18]. Here we evaluated the time-course of Ang-1 and Ang-2 expression in FN patients early in the course of sepsis and explored the diagnostic accuracy of Ang-1 and Ang-2 levels as potential predictors of poor outcome in this clinical setting.

## Methods

### Patient's eligibility criteria

Recruitment of patients took place at the Bone Marrow Transplantation Unit of University of Campinas between March 2008 and March 2009. Patients were included if they fulfilled the following criteria: (1) diagnosis of hematological malignancies, and (2) admission as inpatients for intensive chemotherapy (induction for acute leukemia or high-dose sequential therapy for lymphomas) or hematopoietic stem-cell transplantation (HSCT). Patients were invited to participate before the initiation of any chemotherapy regimen. Fever (T ≥ 38.0°C) at admission was the only exclusion criteria. The study was performed in accordance with the Declaration of Helsinki and approved by the local Ethics Committee. Informed written consent was obtained from all patients prior to collection of samples. Only patients that presented fever during neutropenia (defined as a neutrophil count <500 μl) were included in the second phase of the study. Descriptive data consisting of demographics, diagnosis, clinical data, and disease severity scores were obtained from the medical records. Twenty healthy individuals volunteered to determine a normal reference range for Ang-1 and Ang-2 levels (10 males, 10 females; median age 40 - range 24 to 53).

### Sepsis definitions and risk stratification scores

An infectious etiology was assumed for all patients with post chemotherapy neutropenia with new onset fever, in accordance with FN management protocols. Blood and urine cultures were immediately obtained and broad-spectrum antibiotics were initiated [19]. Sepsis, in this population, was defined by the presence of two or more of the following: (1) temperature > 38.0°C, (2) heart rate>90 beats/min, (3) respiratory rate > 20 breaths/min or PaCO_2 _< 32 mmHg, and a microbiologically proven or clinically evident source of infection. Septic shock was present in patients in which sepsis was complicated with hypoperfusion or hypotension (systolic arterial pressure <90 mmHg or a reduction in systolic blood pressure of >40 mmHg from baseline), despite adequate volume resuscitation. Patients were subdivided into two outcome groups: sepsis (non-complicated) and septic shock. Severity of illness was assessed by calculating the Sequential Organ Failure Assessment (SOFA) score [20] daily after the development of fever, and by calculation of the Multinational Association for Supportive Care In Cancer (MASCC) score at the time of fever [21,22].

### Laboratory measurements

Venous blood was drawn at enrollment (baseline), within 12 hours after first episode of neutropenic fever, and 48 hours thereafter. Samples were immediately centrifuged at 3000 rpm (4°C, 20 mins) and plasma and serum was stored at -80°C until analysis. Samples were processed by the same investigator. Serum levels of Ang-1 and Ang-2 were measured in duplicate using a commercial enzyme-linked immunosorbent assay (ELISA) kit (Quantikine, R&D Systems, Minneapolis, MN, USA) according to the manufacturer's instructions. Interassay coefficient of variations were 2.95% for Ang-1 and 6.87% for Ang-2 samples with concentrations within the range observed in our study. Von Willebrand Factor (VWF) levels were measured in duplicate in citrate plasma samples using a rabbit anti-human VWF peroxidase conjugate (Dako Netherlands BV, Heverlee, Belgium).

### Statistical Analysis

Patients were divided in two outcome subgroups according to the presence of absence of septic shock at any time point before the resolution of FN. Differences in continuous variables between patients from each subgroup, and between patients and healthy controls were analyzed using the Mann-Whitney rank sum test. Categorical variables were compared using the Fisher's exact test. Data are expressed as median and range unless otherwise stated. Correlation (Spearman's rank correlation) and linear regression analysis were performed between sepsis severity scores and angiopoietin concentrations. Receiver operator characteristics (ROC) procedures were used to identify optimal cut-off values of angiopoietin concentrations to differentiate patients with non-complicated sepsis and patients with septic shock. A second analysis was performed to explore the effect of angiopoietin concentrations on 30-day mortality, calculated from the day of fever onset. Survival curves were estimated using the Kaplan-Meier method. Parameters independently associated with survival were identified by univariate and multivariate Cox proportional hazards models. Variables found to be statistically significant at a 10% level in the univariate analyses were included in the multivariate model. Different models were established, and variable selection was performed by different forward and backward procedures, with comparable results. A *p value *less than or equal to 0.05 was considered statistically significant. All statistical analysis were performed with the SPSS package (SPSS Inc., Chicago, IL, USA) and the GraphPad Prism Software (GraphPad Prism Software Inc. San Diego, California, USA).

## Results

### Patients Characteristics

**Figure 1:**
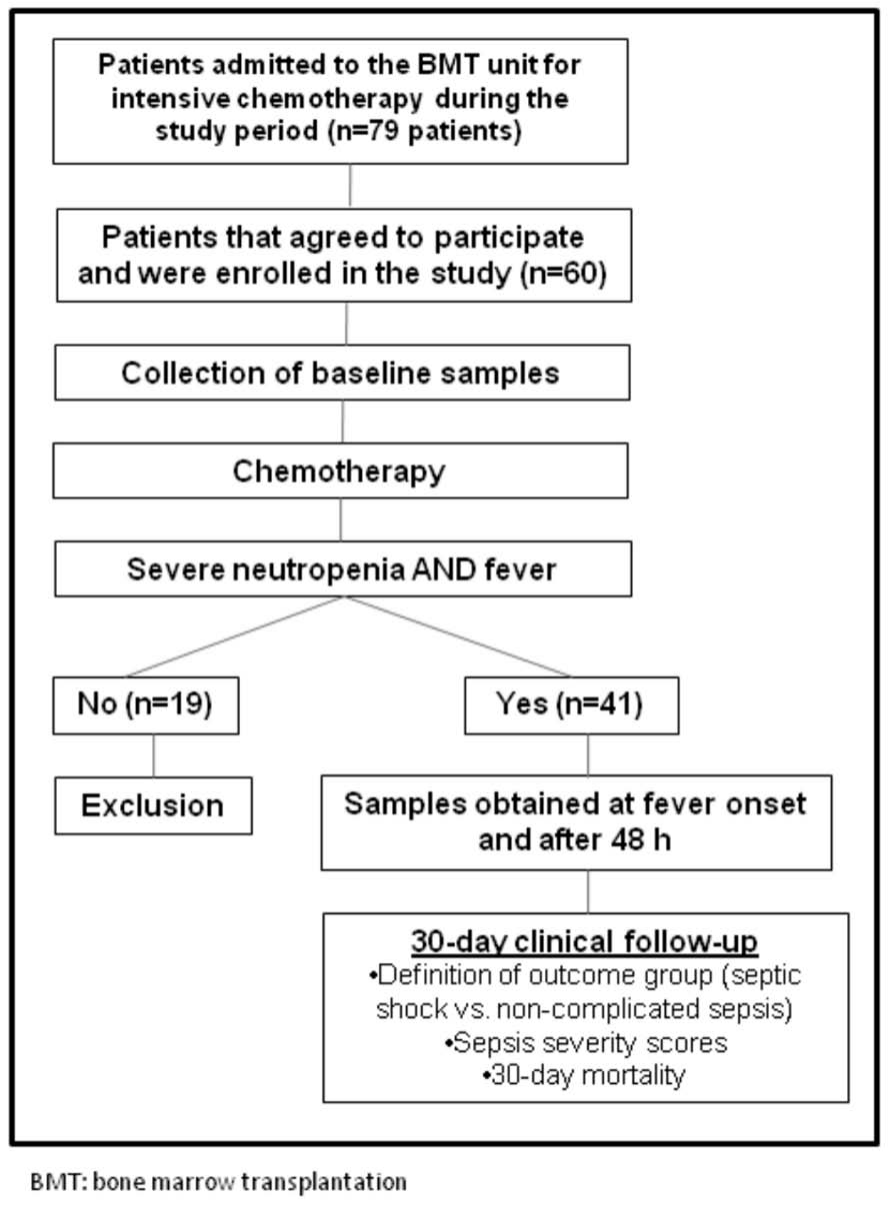
**Flowchart of the study**.

A total of 60 patients fulfilled the primary criteria for study entry, of which 41 patients experienced neutropenic fever and completed the study (Figure 1). Characteristics of these patients are shown in Table 1. In 10/41 patients (24%), septic shock was present before the resolution of neutropenia, and all of them required mechanical ventilation. The median time to the development of septic shock was 4.1 days (range 1 - 7 days) after the first episode of neutropenic fever, and in only one patient, septic shock onset occurred in the first 48 hours after the first episode of neutropenic fever. Eight patients died within the first 30 days after the onset of fever, reaching a 30-day mortality of 13.3%. All deaths were attributed to complications of septic shock. The only clinical significant difference between patients with non-complicated sepsis and septic shock were: (1) age, (2) presence of bloodstream infection, (3) sepsis severity score SOFA calculated 48 hours after fever onset, and (4) MASCC at the time of neutropenic fever. An anatomic site of infection was established in 10 (24%) patients. Compared to healthy individuals, patients presented lower baseline levels of Ang-1, and similar baseline levels of Ang-2 (Table 2).

**Table 1:** Patient characteristics

Characteristics	Sepsis ¥ (n = 31)	Septic shock (n = 10)	P
Sex (male:female)	13:18	7:3	0.16 **

Age (median, range)	37 (16-55)	55 (24-62)	P < 0.01 *

**Diagnosis**			

Acute leukemias	17	4	

Other ^€^	14	6	

**Disease status**			0.12 **

Complete remission	13 (42%)	1 (10%)	

Active disease	18 (58%)	9 (90%)	

**Treatment**			0.48 **

Intensive CTx/autologous HSCT	17	6	

Allogeneic HSCT	14	4	

**Neutrophils/μl - Fever **(median, range)	60 (0 - 290)	50 (20 - 470)	0.40 *

**Days of neutropenia **(median, range)	12 (4 - 22)	14 (7 - 30)	0.36 *

**Platelets × 10^3^/μl - fever **(median, range)	25 (6 - 169)	38 (12 - 90)	0.16 *

**Days with fever **(median, range)	4 (1 - 12)	5 (1 - 12)	0.65 *

**SOFA score - fever onset **(median, range)	3 (0 - 7)	4 (2 - 8 )	0.31 *

**SOFA score - 48 hours **(median, range)	4 (2 - 7)	7 (4 - 16)	P = 0.01 *

**MASCC score **(median, range)	21 (16 - 23)	18 (11 - 24)	P = 0.03 *

**Agent isolation in bloodstream **(yes:no) ^£^	4:27	8:2	P < 0.001 **

**Von Willebrand Factor U/ml - fever **(median, range)	205.12(88.94 -341.95)	262.72 (208.19-430.09)	0.11 *

¥ Non-complicated sepsis; *Mann-Whitney test; **Fisher's exact test; €: Lymphoma (n = 8); Multiple Myeloma (n = 5); Myeloproliferative diseases (n = 5); Paroxysmal Nocturnal Hemoglobinuria (n = 2); HSCT: Hematopoietic stem cell transplantation; CTx: chemotherapy. £: Gram negative: n = 7; Gram positive: 7; Fungi: n = 2 (4 patients with mixed flora infections).

**Table 2:** Baseline levels of Ang-1 and Ang-2 in the study population

	Healthy individuals (N = 20)	Patients (N = 41)	P*
**Ang-1 **(pg/ml)	8799.70 (4795.10-14644.00)	3746.90 (76.43-51780.00)	<0.0001

**Ang-2 **(pg/ml)	41.01 (19.40-246.09)	48.37 (18.81-2892.10)	0.88

	**Sepsis **€ (n = 31)	**Septic shock **(n = 10)	

**Ang-1 **(pg/ml)	4156.60 (100.33-51780.00)	3224.00 (76.43-6117.50)	0.30

**Ang-2 **(pg/ml)	55.22 (19.40-2892.10)	26.04 (18.10-218.60)	0.24

* Mann-Whitney test; € Non-complicated sepsis

### Time-course of Ang-1 serum levels in hematological patients with FN

**Figure 2:**
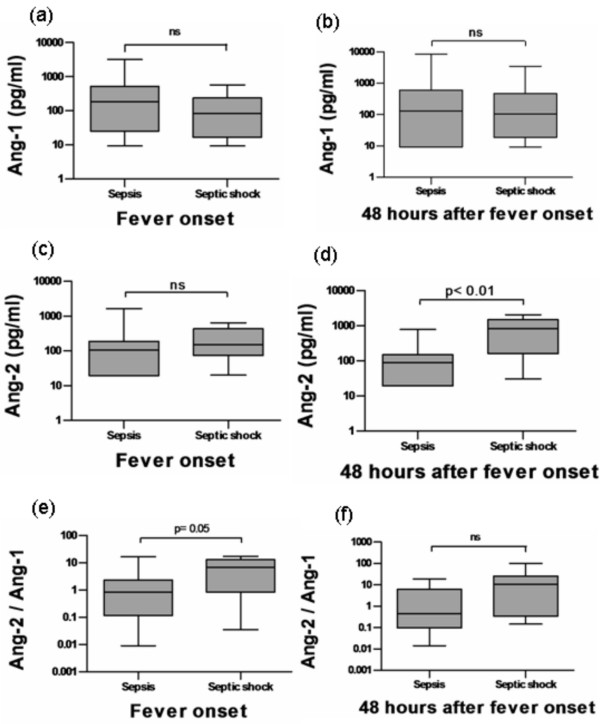
**Serum angiopoietin levels in patients with febrile neutropenia stratified by outcome group**. Box plots representing serial concentrations of angiopoietins in patients with febrile neutropenia that presented non-complicated sepsis (n = 31) or septic shock (n = 10). Ang-1 at the time of fever onset (a) and after 48 hours (b). Ang-2 at the time of fever onset (c) and after 48 hours (d). Ang-2/Ang-1 ratio at the time of fever onset (e) and after 48 hours (f). Horizontal bars indicate median values. Mann-Whitney test.

At baseline, no difference in Ang-1 levels could be observed between patients with non-complicated sepsis and with septic shock (Table 2). No statistical significant difference could be detected between Ang-1 levels in patients with non-complicated sepsis (185.46 pg/ml, range 9.30-3206.40 pg/ml) or with septic shock (82.34 pg/ml, range 9.30-571.05 pg/ml; Mann-Whitney test, p = 0.29) at the time of fever onset. After 48 hours, Ang-1 levels remained similar in patients with non-complicated sepsis (129.27 pg/ml, range 9.30-8485.10 pg/ml) and in patients with septic shock (105.10 pg/ml, range 9.30-3510.60 pg/ml; Mann-Whitney test, p = 0.90) (Figure 2).

### Time-course of Ang-2 serum levels in hematological patients with FN

At baseline, no statistically significant difference could be observed in levels of Ang-2 between patients from the two groups (Table 2). Furthermore, Ang-2 levels were similar at the time of neutropenic fever between patients with non-complicated sepsis (105.55 pg/ml, range 19.40-1653.50 pg/ml) and septic shock (152.39 pg/ml, range 19.97-644.27 pg/ml). However, 48 hours after neutropenic fever a striking difference could be observed between patients with and without septic shock, with markedly increased Ang-2 concentrations in patients with septic shock (840.77 pg/ml, range 30.67-2085.30 pg/ml) compared to patients with non-complicated sepsis (91.10 pg/ml, range 19,40-785,24 pg/ml; Mann-Whitney test: p = 0.002) (Figure 2). We estimated the diagnostic accuracy of Ang-2 levels measured 48 hours after fever onset. Ang-2 48 hours after fever onset yielded an area under the ROC curve of 0.84 (95%CI = 0.65-1.0; P = 0.004). In our population, an optimal cut-off value of Ang-2>233 pg/ml predicted the development of septic shock with a sensitivity of 75% (95%CI = 34.9%-96.8%) and a specificity of 92.6%(95%CI = 75.7%-99.1%).

### The Ang-2/Ang-1 ratio is increased in FN patients that evolve to septic shock

The Ang-2/Ang-1 ratio was calculated in an effort to detect relevant changes in the relative concentration of these two antagonistic mediators of microvascular permeability. At the time of neutropenic fever, the Ang-2/Ang-1 ratio was much higher in patients with septic shock (6.80, range 0.03-17.20) compared to patients with non-complicated sepsis (0.80, range 0.01-16.8; Mann-Whitney test: p = 0.05). After 48 hours, the Ang-2/Ang-1 ratio was higher in patients that developed septic shock (10.58, range 0.10-101.70) compared to patients with non-complicated sepsis (0.40, range 0.01-18.70), but statistical significance was not reached (Mann-Whitney test: P = 0.06) (Figure 2). Estimation of the diagnostic accuracy of the Ang-2/Ang-1 ratio at fever onset yielded an area under the ROC curve of 0.71 (95%CI = 0.50-0.93; P = 0.05). In our population, a median Ang-2/Ang-1 ratio of 1.17, which was the optimal cut-off value identified by the ROC procedure, predicted the development of septic shock with a sensitivity of 77.8% (95%CI = 40.0%-97.2%) and a specificity of 60% (95% CI = 40.6%-77.3%).

### Serum Ang-1 and Ang-2 levels correlate with severity of illness score (SOFA)

**Figure 3:**
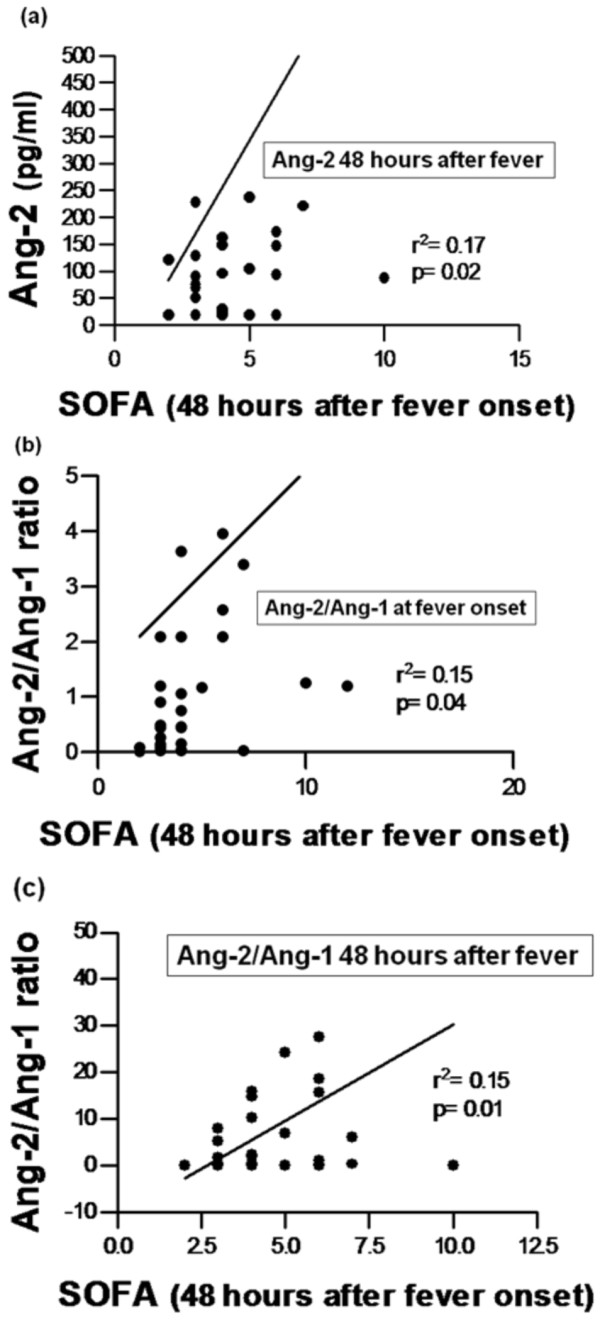
**Correlation of angiopoietin levels with the SOFA sepsis severity score**. Scatter plots showing linear regression analysis between Sequential Organ Failure Assessment (SOFA) scores and (a) Ang-2 concentrations measured 48 h after fever onset, (b) Ang-2/Ang-1 ratio measured at fever onset (b) and 48 hours thereafter (c).

We next evaluated whether serum Ang-1 and Ang-2 levels correlated with the SOFA score. We utilized the SOFA score calculated 48 hours after fever because it segregates each outcome group better than SOFA at the time of fever onset (Table 1). Significant correlations were observed between SOFA and the following parameters: Ang-2 48 h after fever (Rs = 0.40; P = 0.02), Ang-2/Ang-1 ratio at fever onset (Rs = 0.51; P = 0.001) and Ang-2/Ang-1 ratio 48 h after fever (Rs = 0.35; P = 0.04) (Figure 3).

### Ang-2/Ang-1 ratio at fever onset and 30-day mortality in patients with FN

**Figure 4:**
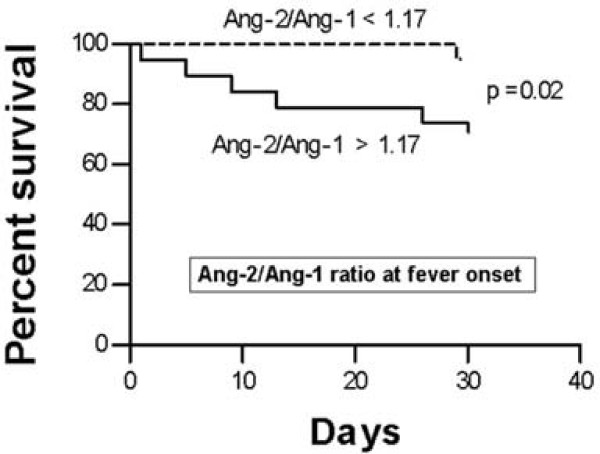
**Survival of patients with febrile neutropenia according to Ang-2/Ang-1 ratio at the time of fever onset**. Kaplan-Meier curve (less versus greater than median; * Logrank test.

To determine the relationship of angiopoietin levels with 30-day mortality, we initially performed univariate Cox proportional hazards analysis, in which the following variables were found to be statistically significant: age, duration of neutropenia, SOFA, MASCC, Ang-2 (48 hours after fever) and Ang-2/Ang-1 ratio (both at fever onset and 48 hours thereafter). We then performed multivariate Cox regression analysis incorporating these variables, and the only variable that remained statistically significant in the multivariate setting was the Ang-2/Ang-1 ratio, measured at fever onset (Hazard ratio 1.20 - 95%CI 1.02-1.41; P < 0.01). Figure 4 illustrates the Kaplan-Meier curve of 30-day survival stratified to less versus greater than the median values of Ang-2/Ang-1 ratio at fever onset. Logrank confirmed statistical significance. The 30-day mortality of patients with Ang-2/Ang-1 ratio below 1.17 was 5.5% compared to 31.6% for patients with greater ratios.

## Discussion

Post chemotherapy FN in patients with hematological malignancies is a condition that carries a high risk of sepsis complications with mortality rates as high as 21% [16], usually preceded by septic shock. Patients with FN are a heterogeneous group in terms of risks of complications and mortality, and the identification of parameters capable to accurately identify high risk patients is one of the great challenges in their care. Clinical scores such as the MASCC [21], and laboratory parameters such as C-reactive protein, IL-6, IL-8 and procalcitonin have been recently proved to be useful tools for this purpose [23]. However, it is generally acknowledged that these parameters are more useful to identify patients at low risk for complications, leaving room for refinements in the risk stratification of FN patients. Ideally, one such marker should be easily detected in samples obtained before the development of severe sepsis, and should not be influenced by cytopenias or by the inflammatory milieu associated with disease status. Elements that are directly involved in the pathogenesis of sepsis complications are thus more attractive candidates than non-specific elements of the inflammatory cascade.

Angiopoietins are a family of vascular growth factors with critical roles in embryonic and postnatal angiogenesis. Ang-1 and Ang-2 both act on the Tie2 tyrosine kinase receptor found primarily on endothelial cells, but appear to play antagonist roles [10]. Apparently, Ang-1 promotes vessel stabilization whereas Ang-2 is involved in the destabilization of newly formed vessels. These processes of disassemble and reassemble of the endothelial lining of primitive blood vessels is important because it allows the incorporation of new endothelial cells to growing endothelial tubes, leading eventually to the formation of functional and mature blood vessels [24]. A role of angiopoietins in the pathogenesis of septic shock is supported by multiple lines of evidence. First, increased levels of Ang-2 levels have been demonstrated in adult and pediatric patients with sepsis in intensive care units (ICU) and these levels correlated with sepsis severity [11,12,14,25]. Second, serum from patients with sepsis has been shown to disrupt endothelial architecture, an effect that correlates with Ang-2 levels, is reversed by Ang-1 and is mimicked by recombinant Ang-2 [14]. Finally, Ang-2 levels have been shown to correlate with pulmonary permeability edema and occurrence of acute respiratory distress syndrome in mechanically ventilated patients [26]. More recently, Kumpers et al, studying a population of 43 medical ICU patients demonstrated that Ang-2 levels at the time of ICU admission was a strong predictor of mortality [12]. Ang-1 levels were also evaluated in some of these studies, but so far studies have failed to a relationship between Ang-1 and sepsis outcomes.

In our prospective study we explored the time-course of Ang-1 and Ang-2 in a population of patients with high risk of sepsis complications before the development of severe sepsis. As far as we are aware, the significance of Ang-1 and Ang-2 levels has not been studied in patients with FN. An additional contribution of our study is that we prospectively evaluated the significance of Ang-1 and Ang-2 levels at an earlier time-point in the development of sepsis (which developed in a median time of 4.1 days after fever onset), as opposed to studies that evaluated patients at the ICU. This difference is evidenced by the median SOFA score of our patients (4 range 1-8) compared to higher median SOFA scores (16 range 1-22) from other key studies [12]. Last, Ang-1 and Ang-2 levels were serially evaluated at three time points, thus offering a view of the time-course of Ang-1 and Ang-2 release in the early hours of sepsis.

For this study we only included patients with hematological malignancies and FN after intensive chemotherapy that were treated as inpatients from day one of chemotherapy until the resolution of neutropenia. By doing so, we intended to obtain a representative sample of patients with high risk of sepsis complications. Furthermore, the fact that all patients were treated as inpatients allowed us to standardize important variables that could affect the validity of our results such as the time between fever onset and sample collection. So as to limit as much as possible the influence of different diagnosis and chemotherapy regimens on our results, Ang-1 and Ang-2 levels were also collected immediately before the initiation of chemotherapy, allowing differences between outcome groups to be compared not only as absolute values, but also as fold-increase from baseline levels, which did not alter our results (data not shown). In fact, neither Ang-1 nor Ang-2 levels were significantly different before the initiation of chemotherapy between patients with non-complicated sepsis and septic shock, supporting the uniformity of our group of patients as far as the two study variables were concerned.

The main finding of our study is that the relative concentration of Ang-1 and Ang-2 are different in subgroups of patients with FN that evolve to non-complicated sepsis compared to patients that develop septic shock, and that evaluation of these two proteins within the first 48 hours after neutropenic fever, before the development of any signs and symptoms of septic shock, is a promising tool to discriminate high risk patients with FN. In accordance with previous studies, Ang-2 levels were significantly higher in patients with septic shock compared to patients with non-complicated sepsis. This difference was not present at the time of fever onset, rose sharply and became evident after 48 hours, when levels in the poorer outcome group were 8 times higher than in the good outcome group. Importantly, Ang-2 baseline levels were similar. As in previous studies, we were not able to detect any significant difference of Ang-1 levels between study groups. However, when the relative concentrations of these two antagonistic cytokines were evaluated, we did observe a relative deficiency of Ang-1 compared to Ang-2 levels in patients that developed septic shock, that was evident early at the time of fever onset. It has been known for more than a decade that Ang-1 can protect adult vasculature against VEGF-induced plasma leakage [10]. Ang-1 has also been shown to protect mice from endotoxic shock [27]. In this respect, it is tempting to speculate that the imbalance between Ang-1 and Ang-2 levels present already at the time of fever onset is associated with a poorer outcome of sepsis in FN, and possibly in other patients with sepsis. This hypothesis is well illustrated in our work by the divergent trend of Ang-2/Ang-1 ratio observed in each outcome subgroup of patients. Early in the course of sepsis, patients that evolved to septic shock presented a 7-fold increase in the Ang-2/Ang-1 ratio, whereas patients with non-complicated sepsis presented a 0.8 decrease when compared to baseline (pre-chemotherapy) ratios. After 48 hours, this divergent trend persisted, with patients that evolved to septic shock presenting a 10-fold increase, and patients with non-complicated sepsis with a 0.4-fold decrease. We also evaluated VWF antigen levels, which is increased in patients with sepsis [28], and is an indicator of endothelial dysfunction and stimulation [29]. In contrast to Ang-2, no difference could be observed between both patient groups at any time-point (table 1). We also demonstrated a significant, though weak correlation of Ang-2 concentrations 48 hours after fever onset and of Ang-2/Ang-1 ratio (both at fever onset and after 48 hours) with the SOFA score of sepsis severity. Based on these results, we estimated diagnostic accuracy of Ang-2 concentrations 48 hours after fever and of Ang-2/Ang-1 ratio at fever onset. Despite wide confidence intervals, area under the ROC curves suggest that both biomarkers should be further investigated as promising tools to discriminate high-risk FN patients in studies with larger sample sizes. The relationship between angiopoietin concentrations and 30-day mortality was also evaluated. Using a multivariate Cox model, we demonstrate that the Ang-2/Ang-1 ratio at the time of fever onset can be an independent predictor of sepsis survival in our population, an observation that will have to be validated in larger studies.

Recently, the time course of Ang-1 and Ang-2 release was evaluated in a model of human endotoxemia [13], which showed that Ang-2 release is initiated 2.5 hours after LPS challenge, and observed no significant variations in levels of Ang-1. Ang-2 is a Weibel-Palade body-stored molecule that is rapidly released upon endothelial stimulation. In contrast, Ang-1 is believed to exert its vessel-sealing effect by low-level constitutive activation of the Tie2 receptor, in a model in which constitutive Ang-1/Tie2 interactions control endothelial barrier integrity as a default pathway, and Ang-2 acts as a dynamically regulated antagonizing cytokine [30]. Our results showing that patients that evolve to septic shock present an initial relative deficiency of Ang-1 associated with a sharp increase in Ang-2 levels in the first 48 hours of sepsis supports that similar events can be involved in the pathogenesis of septic shock in FN.

There are certain limitations of our study that need to be acknowledged to avoid overinterpretation of its conclusions. Research about new diagnostic tools follow a sequence of phases along which the questions answered by each phase progress from the demonstration that a biomarker behaves differently in diseased and normal individuals (phase I) to the ultimate demonstration of improved clinical outcomes after its incorporation (phase IV) [31]. Ours is a phase II study in which the performance of a new diagnostic assay was tested in patients with a defined condition (febrile neutropenia) and potential different outcomes, but in which several confounding variables were controlled. Phase II studies tell us whether the test shows diagnostic promise under ideal conditions. Therefore, our study design does not allow us to universalize our conclusions to clinical settings where the impact of treatment related and other variables might reject our conclusions. For the formal validation of the assay and its incorporation intro clinical practice, a larger sample size (ideally from distinct centers) under less controlled conditions should be used. The relatively low number of deaths used for the multivariate analysis also deserves to be discussed. Our study design required a very specific population of patients, with stringent inclusion criteria to control confounding variables, so that a further increase in sample size was not feasible. On the other hand, it has been demonstrated that relaxing the rule of 10 events per variable in Cox models is possible without unacceptable increases in confounding bias [32]. With this in mind, we believe that as long as the above-mentioned limits of our study are acknowledged, the association of 30-day mortality with Ang-2/Ang-1 ratio is an important information to the literature.

## Conclusions

In summary, our data suggest that imbalances in the concentrations of Ang-1 and Ang-2 present already at the time of the first fever peak is an independent marker of the risk of developing septic shock and of 30-day mortality in FN. Further studies are warranted to validate this new biomarker as clinically relevant tool in the daily care of these patients. In addition, therapeutic strategies designed to manipulate the Ang-2/Ang-1 imbalance can offer a new and promising paradigm for the treatment of sepsis and septic shock in patients with FN.

## Competing interests

The authors declare that they have no competing interests.

## Authors' contributions

BEA enrolled patients, recorded clinical data, performed laboratory analysis and contributed to manuscript production; SALM and TMGS performed laboratory analysis; FJPA performed statistical analysis and reviewed the manuscript; CAS, IL and JMA contributed to the study design and reviewed the manuscript; EVDP designed the study, analyzed data and contributed to manuscript production. All authors read and approved the final manuscript.

## Pre-publication history

The pre-publication history for this paper can be accessed here:

http://www.biomedcentral.com/1471-2334/10/143/prepub
